# Control of chemotherapy-induced nausea in patients receiving outpatient cancer chemotherapy

**DOI:** 10.1007/s10147-015-0908-2

**Published:** 2015-10-16

**Authors:** Hirotoshi Iihara, Hironori Fujii, Chiaki Yoshimi, Maya Yamada, Akio Suzuki, Nobuhisa Matsuhashi, Takao Takahashi, Kazuhiro Yoshida, Yoshinori Itoh

**Affiliations:** Department of Pharmacy, Gifu University Hospital, Gifu, Japan; Department of Surgical Oncology, Gifu University Graduate School of Medicine, Gifu, Japan

**Keywords:** Outpatient cancer chemotherapy, Nausea, Emetic risk, Risk analysis, Adherence to guideline

## Abstract

**Background:**

Control of chemotherapy-induced nausea is still incomplete, regardless of adherence to the antiemetic guideline. The present study was designed to assess the control rates of nausea and vomiting in the outpatient chemotherapy clinic and to determine risk factors for nausea.

**Methods:**

A single-center prospective observational study was carried out in 779 patients who received 5511 chemotherapy cycles from January 2013 to December 2014 in the outpatient chemotherapy clinic. A checklist for adverse events was provided to all patients, and nausea and vomiting were monitored on the next visit. Complete protection from nausea and vomiting during acute (within 24 h) and delayed (during 2–7 days) periods was assessed.

**Results:**

Under the condition of 76–99 % rates of adherence to the Japanese Society of Clinical Oncology guideline for antiemesis, the rates of complete protection from acute and delayed nausea in the first cycle of chemotherapy were 60 % and 45 %, respectively, for high emetic risk chemotherapy (HEC), and 85 % and 70 % for moderate emetic risk chemotherapy (MEC). The rates were improved in the overall cycles. On the other hand, vomiting was well controlled, in which complete protection ranged from 83 % (HEC) to 99 % (minimum). A multivariate analysis indicated that being female, age less than 60 years, high or moderate risk chemotherapy, and anthracycline/cyclophosphamide (A/C) were significant risks for overall nausea. Indeed, the control of delayed nausea was extremely poor in the first cycle of A/C, although there was no difference in the control of nausea among MEC.

**Conclusion:**

Antiemetic medication in consideration of the risk factors is required to improve the control of nausea.

## Introduction

Chemotherapy-induced nausea and vomiting (CINV) were the first and second most distressing adverse events in the past [1]. Nausea and vomiting severely impair the patient’s quality of life, which may lead to the discontinuance of therapy. However, the rank order of nausea and vomiting has dropped [2] since the development of 5-hydroxytryptamine-3 (5-HT_3_) receptor antagonists and the prevalence of the American Society of Clinical Oncology (ASCO) clinical practice guideline for antiemesis [3]. Subsequently, with the development of novel classes of antiemetic drugs such as neurokinin 1(NK_1_) receptor antagonists, including aprepitant, and palonosetron, a second-generation 5-HT_3_ receptor antagonist, the antiemetic guideline has been revised by several international societies for clinical oncology, including ASCO [4], Multinational Association of Supportive Care in Cancer (MASCC) [5], National Comprehensive Cancer Network (NCCN) [6], and Japanese Society of Clinical Oncology (JSCO) [7].

However, guideline-consistent antiemetic medication is not always widely used in clinical practice, a so-called evidence–practice gap [8]. Gomez et al. [9] reported, in patients who received high emetic risk chemotherapy (HEC) or moderate emetic risk chemotherapy (MEC), that the use of 5-HT_3_ receptor antagonist with dexamethasone is 60–90 %, whereas the use of a NK_1_ antagonist is much less common (<10 %). Hori et al. [10] also reported by a nationwide survey of 9978 patients receiving 81,739 chemotherapy cycles from 39 Japanese hospitals that the rates of adherence to the JSCO guideline during acute and delayed periods are 28.1 % and 9.7 %, respectively, for HEC, and 7.2 % and 6.9 %, respectively, for MEC.

It has been demonstrated that adherence to the antiemetic guideline improves the control of CINV [11–13]. Chan et al. [11] reported in 361 breast cancer patients receiving anthracycline-based chemotherapy that the adherence to the antiemetic guideline is 57.9 % and that the complete control of CINV is better in the guideline-consistent group than in the guideline-inconsistent group, indicating that adherent patients were more likely to achieve complete control of CINV [adjusted odds ratio (OR) 1.74, 95 % confidence interval (CI) 1.01–3.01, *P* = 0.048]. Aapro et al. [12] also reported in 991 patients receiving the first cycle of HEC or MEC that the complete response (no vomiting and no rescue) is significantly better in the guideline-consistent group than in the guideline-inconsistent group (59.9 % vs. 50.7 %, *P* = 0.008), resulting in the adjusted odds ratio of 1.43 (95 % CI 1.04–1.97, *P* = 0.027). We also previously reported in 125 colorectal cancer patients receiving the first cycle of MEC such as levofolinate, fluorouracil, oxaliplatin (mFOLFOX6) and levofolinate, fluorouracil, irinotecan (FOLFIRI) regimen that the complete control of nausea but not vomiting is significantly better in the guideline-consistent group than in the guideline-inconsistent (lack of dexamethasone on days 2 and 3) group (74 % vs. 56 % for nausea, *P* < 0.05; 94 % vs. 91 % for vomiting) [14].

On the other hand, cancer chemotherapy is currently shifting from inpatient admission to the outpatient setting. Chemotherapy-induced vomiting is almost preventable in the outpatient setting by the addition of NK_1_ receptor antagonists and 5-HT_3_ receptor antagonists to the standard medication, although nausea remains a distressing adverse drug reaction [15, 16]. In the present study, the antiemetic medication consistent with the JSCO guideline has been implemented in our outpatient chemotherapy clinic, and the rates of complete protection of CINV were subsequently investigated. Risk analysis for overall nausea was also carried out.

## Patients and methods

### Patients

There were 9590 visits for cancer chemotherapy in our outpatient chemotherapy clinic during the 2 years from January 2013 to December 2014; among these, chemotherapy was carried out in 8206 visits, thereby indicating a 14.4 % discontinuance rate. The actual number of patients counted by the patient ID number was 779 and the number of chemotherapy cycles was 5577. Health professionals such as pharmacists and nurses were in charge of provision of drug information and safety precaution in daily life and of monitoring adverse drug reactions to all patients in our outpatient chemotherapy clinic. Patient consultation was carried out in 8206 visits (100 %), but the CINV data were obtained from 5511 chemotherapy cycles. Thus, data from 98.8 % of the chemotherapy cycles were subjected to analysis in the present study.

The present study was carried out in accordance with the guidelines for the care for human study adopted by the Ethics Committee of the Gifu Graduate School of Medicine, and notified by the Japanese Government (approved no. 26-153 of the Institutional Review Board).

### Adherence to the Japanese guideline for the use of antiemetic drugs

According to the JSCO clinical practice guideline for antiemesis, the use of antiemetic drugs was promoted for prevention of CINV: the combination of 5-HT_3_ receptor antagonist, aprepitant, and dexamethasone was used before chemotherapy and a combination of aprepitant and dexamethasone was provided on days 2 and 3, and dexamethasone on day 4 for high emetic risk chemotherapy (HEC), a combination of 5-HT_3_ receptor antagonist and dexamethasone was taken before chemotherapy, and dexamethasone was prescribed on days 2 and 3 for moderate emetic risk chemotherapy (MEC), and dexamethasone was administered only before chemotherapy for low emetic risk chemotherapy. No routine antiemetic drug was provided for prevention of minimum risk chemotherapy. Health professionals made extensive efforts in facilitating the use of appropriate antiemetic premedication by inclusion of antiemetic regimens into the prescription order for chemotherapy regimens or by proposing the prescription of antiemetic drugs to physicians. Adherence to the Japanese antiemetic guideline in both acute and delayed periods was evaluated. In the case of the dose of dexamethasone, the guideline recommends 8 mg/day in the delayed period for HEC and MEC. In the present study, however, 4 mg/day on days 2–3 for MEC was regarded as positive for adherence because previous findings indicated that the control of delayed nausea is improved by 20 % by the addition of 4 mg/day of dexamethasone on days 2–3, when compared with no treatment with dexamethasone in patients receiving MEC [14].

### Evaluation of chemotherapy-induced nausea

All patients were provided with a checklist for daily check of adverse events on their first visit to the outpatient chemotherapy clinic. Using the checklist, patients checked daily their nausea by numeric rating scale (0–10) and the number of vomiting episodes up to 7 days after chemotherapy. Pharmacists and nurses recorded the control of nausea and vomiting on the electronic medical record after verifying the data or hearing results from patients on the next visit. Complete protection from nausea (NRS scale <1) and vomiting (no episode) during acute (within 24 h after chemotherapy) and delayed (during 2–7 days after chemotherapy) periods was assessed in patients receiving the first cycle of chemotherapy or in those with overall cycles of chemotherapy.

### Risk analysis for chemotherapy-induced nausea during overall period

Demographics of patients were compared between patients who revealed acute and delayed nausea and those who showed complete protection from nausea. Subsequently, uni- as well as multivariate logistic regression analyses were carried out to determine the risk for incomplete protection from for nausea or vomiting during the overall period. Odds ratio (OR) and 95 % confidence interval (CI) were determined. The cutoff value of age was determined by the Youden index or the distance method in the receiver operating characteristic curve (ROC) analysis. In the Youden index, cutoff age was estimated from the maximum value of (sensitivity + specificity − 1), whereas in the distance method, cutoff value was predicted from the minimum value for square root [(1−sensitivity)^2^ + (1−specificity)^2^)], according to the method described earlier [17, 18].

### Statistical analyses

Data were analyzed using IBM SPSS Statistics ver. 22 (IBM Japan Services, Tokyo, Japan) and Graph Pad Prism version 6.0 (Graph Pad Software, San Diego, CA, USA). Parametric variables were analyzed using the *t* test, and nonparametric data were analyzed by the Mann–Whitney *U* test or the chi-square test. Multiple comparisons for the control of nausea and vomiting were carried out by Kruskal–Wallis test followed by Scheffe’s test. A *P* value less than 0.05 was considered statistically significant.

## Results

### Demographics of patients

Demographics of the patients are shown in Table 1. Seven hundred and seventy-nine patients received 5511 chemotherapy cycles during 2 years from January 2013 to December 2014 in our outpatient chemotherapy clinic. The average chemotherapy cycle was 8.3 cycles. The most common type of cancer was colorectal cancer (26 % of patients and 35 % of all chemotherapy cycles), followed by lung cancer (14 % and 12 %), breast cancer (14 % and 17 %), gastric cancer (13 % and 12 %), liver/gallbladder/pancreas cancer (10 % and 9 %), hematological cancer (9 % and 5 %), gynecological cancer (6 % and 5 %), and head and neck cancer (3 % and 2 %). The emetic risk of the chemotherapy regimens included HEC (291 cycles, 5 %), MEC (2184 cycles, 40 %), low risk (2162 cycles, 39 %), and minimum risk (874 cycles, 16 %).

**Table 1:** Demographics of patients

Number of patients (male/female)	779 (391/388)
Age (average, min/max)	63.4 (18/88)
Body surface area (average, SD)	1.56 ± 0.40
Serum creatinine (mg/dl, average, SD)	0.72 ± 0.27
Number of chemotherapy courses	5511

Cancer type	Number of patients	%	Number of courses	%
Colorectal	201	25.8	1923	34.9
Lung	107	13.7	645	11.7
Breast	105	13.5	935	17.0
Gastric	101	13.0	680	12.3
Liver/gallbladder/pancreas	81	10.4	510	9.3
Hematological	73	9.4	270	4.9
Gynecological	49	6.3	259	4.7
Head and neck	26	3.3	117	2.1
Esophageal	15	1.9	49	0.9
Urological	10	1.3	89	1.6
Brain	9	1.2	25	0.5
Sarcoma	1	0.1	1	0.02
Dermatological	1	0.1	8	0.1
Average chemotherapy courses (min/max)	8.3 (1/91)			

Emetic risk of chemotherapy	*n*	%
High	291	5.3
Moderate	2184	39.6
Low	2162	39.2
Minimum	874	15.9

### Control of nausea and vomiting

**Fig. 1:**
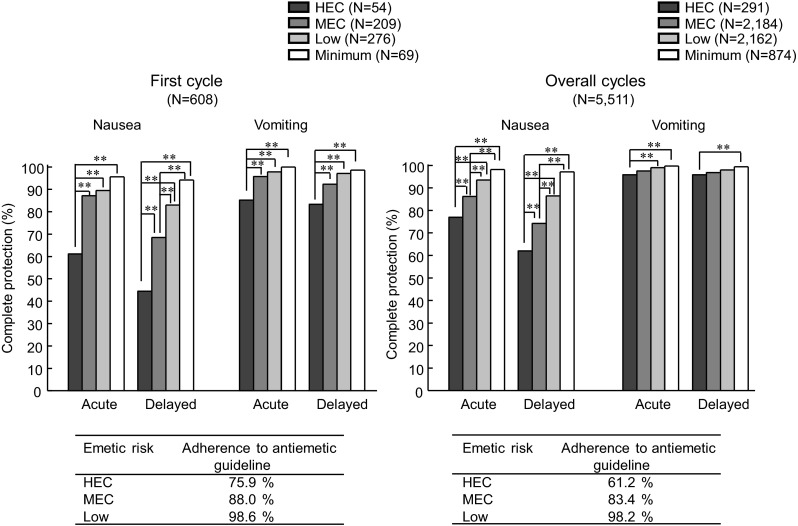
Complete protection from nausea and vomiting during acute and delayed periods in patients receiving high emetic risk chemotherapy (*HEC*), moderate emetic risk chemotherapy (*MEC*), *low* risk, or *minimum* risk of chemotherapy as the first cycle or the overall cycles in the outpatient chemotherapy clinic. Adherence to the Japanese antiemetic guideline is shown at *bottom*. **P < 0.01 by Kruskal–Wallis test followed by Scheffe’s test

The rates of complete protection from nausea and vomiting during acute and delayed periods in the first cycle and overall cycles are shown in Fig. 1. In the first cycle of chemotherapy, the rate of adherence to the Japanese clinical practice guideline for prevention of CINV ranged from 76 % (HEC) to 99 % (low risk). Under such a condition, patients receiving HEC showed poor control of nausea during acute and delayed periods, although vomiting was favorably controlled (83–85 % of complete protection). The rates of complete protection from acute and delayed nausea were 61 % and 44 %, respectively, for HEC, and 87 % and 68 %, respectively, for MEC, in which the rates increased in a manner dependent on the emetic risk of the chemotherapy. In the overall cycles, the control of nausea in HEC was greatly improved, in which the complete protection from acute and delayed nausea was 77 % and 62 %, respectively.

### Comparison of demographics between patients who showed no nausea and those with nausea

To determine the risk of chemotherapy-induced nausea, the demographics of patients were compared between those with and without complete protection from nausea in 608 patients who received the first cycle of chemotherapy. As shown in Table 2, significant differences in gender, age, proportion of HEC/MEC and anthracycline/cyclophosphamide (A/C) regimen, and type of cancer were observed between the two groups. Females were more often affected (67 % vs. 48 %, *P* < 0.001), patient age was younger (56.7 vs. 63.4 years), and the proportion of A/C regimen (18 % vs. 2 %, *P* < 0.001) and HEC/MEC (64 % vs. 36 %) in patients without complete protection from nausea. Moreover, the percentage of breast cancer patients was significantly higher (31 % vs. 18 %, *P* < 0.001) in patients with nausea than those without it.

**Table 2:** Comparison of demographics of patients with nausea during overall period in 608 patients who received the first cycle of chemotherapy

	With nausea during overall period (*n* = 158)	Without nausea during overall period (*n* = 450)	*P*
Ratio of female (female/male)	67.1 (106/52)	47.8 (215/235)	<0.001^a^
Age (years)	56.7 (18–84)	63.4 (35–88)	<0.001^a^
Height (cm)	160.1 ± 7.5	160.3 ± 8.5	0.775^c^
Body weight (kg)	55.0 ± 10.7	55.8 ± 10.8	0.464^c^
Serum creatinine (mg/dl)	0.69 ± 0.23	0.72 ± 0.23	0.170^c^

	*n*	%	*n*	%	*P*
Regimens/anticancer drug					
A/C	28	17.7	9	2.0	<0.001^a^
CHOP	3	1.9	5	1.1	0.733^a^
Oxaliplatin	29	18.4	61	13.6	0.183^a^
Irinotecan	17	10.8	35	7.8	0.323^a^
Carboplatin	11	7.0	29	6.4	0.969^a^
Cyclophosphamide	9	5.7	5	1.1	<0.001^a^
Cisplatin (25–30 mg/m^2^)	2	1.3	14	3.1	0.338^a^
Emetic risk					
HEC + MEC	101	63.9	162	36.0	<0.001^a^
Low + minimum	57	36.1	288	64.0	
Cancer type					
Breast	49	31.0	81	18.0	<0.001^a^
Colorectal	42	26.6	100	22.2	0.315^a^
Lung	6	3.8	82	18.2	<0.001^a^
Gastric	23	14.6	63	14.0	0.968^a^
Liver/gall bladder/pancreas	10	6.3	48	10.7	0.150^a^
Hematological	6	3.8	17	3.8	1.000^a^
Gynecological	16	10.1	26	5.8	0.095^a^
Adherence to antiemetic guideline	146	92.4	420	93.3	0.831^a^

^a^Chi-square test

^b^Mann–Whitney *U* test

^c^ *t* test

On the other hand, adherence to the antiemetic guideline was not different between the two groups (92.4 % vs. 93.6 %, *P* = 0.755).

### Risks for chemotherapy-induced overall nausea or vomiting

Because age was significantly different between patients with and without overall nausea, an ROC curve was plotted for sensitivity versus 1−specificity. The area under the curve (AUC) was 0.658 (95 % CI, 0.607–0.709), indicating low accuracy prediction [17]. Using the ROC curve method, the cutoff age was predicted to be 58.5 years old (Youden index, 72.4 % sensitivity vs. 55.9 % specificity) or 61.5 years old (distance method, 63.6 % sensitivity vs. 62.7 % specificity). Thus, the cutoff age was set to 60 years for nausea. For vomiting, AUC of ROC curve was 0.721 (95 % CI, 0.629–0.813), indicating moderate accuracy prediction [17]. ROC analysis indicated that the cutoff age was 49.5 years old (Youden index as well as distance method, 84.5 % sensitivity vs. 56.4 % specificity). Thus, the cutoff age was set to 50 years old for vomiting.

As presented in Table 3, multivariate analysis showed that four factors such as female gender (OR 1.615; 95 % CI, 1.022–2.552; *P* = 0.004), age under 60 years (OR 2.303; 1.525–3.477; *P* < 0.001), inclusion of HEC/MEC (OR 2.321; 1.489–3.617; *P* < 0.001), and A/C regimen (OR 4.955; 1.863–13.18; *P* = 0.001) were significant risks for overall nausea.

**Table 3:** Risk analysis for nausea and vomiting in 608 patients who underwent the first cycle of cancer chemotherapy

	Univariate analysis			Multivariate analysis		
	Odds ratio (OR)	95 % confidence interval	*P*	OR	95 % confidence interval	*P*
Nausea						
Female	2.228	(1.524–3.258)	<0.001	1.615	(1.022–2.552)	0.040
Age < 60 years	3.007	(2.070–4.369)	<0.001	2.303	(1.525–3.477)	<0.001
HEC/MEC	3.150	(2.160–4.595)	<0.001	2.321	(1.489–3.617)	<0.001
A/C regimen	10.554	(4.857–22.93)	<0.001	4.955	(1.863–13.18)	0.001
Breast cancer	2.048	(1.354–3.098)	0.001	0.700	(0.375–1.306)	0.262
Lung cancer	0.177	(0.076–0.415)	<0.001	0.301	(0.125–0.725)	0.007
Adherence to antiemetic guideline	0.869	(0.434–1.742)	0.692	0.960	(0.444–2.076)	0.918
Vomiting						
Female	4.429	(1.923–10.20)	<0.001	3.151	(1.213–8.183)	0.018
Age < 50 years	4.026	(1.997–8.117)	<0.001	5.803	(2.667–12.63)	<0.001
HEC/MEC	4.152	(1.985–8.683)	<0.001	2.993	(1.245–7.195)	0.014
A/C regimen	8.205	(3.684–18.27)	<0.001	2.987	(0.785–11.36)	0.108
Breast cancer	2.777	(1.420–5.427)	0.003	0.527	(0.167–1.667)	0.276
Lung cancer	0.304	(0.072–1.283)	0.105	0.759	(0.164–3.506)	0.724
Adherence to antiemetic guideline	0.883	(0.260–2.997)	0.842	0.539	(0.138–2.110)	0.375

On the other hand, female gender (OR 3.151; 95 % CI, 1.213–8.183; *P* = 0.018), age under 50 years (OR 5.803; 2.667–12.63, *P* < 0.001), and inclusion of HEC/MEC (OR 2.993; 1.245–7.195; *P* = 0.014) were found to be significant risks for overall vomiting by multivariate analysis. Adherence to the antiemetic guideline did not reduce the risk for overall nausea or vomiting (OR 0.960; 0.444–2.076, *P* = 0.918 for nausea; OR 0.539; 0.138–2.110, *P* = 0.375 for vomiting).

### Comparison of the control of nausea and vomiting among various HEC and MEC regimens

**Fig. 2:**
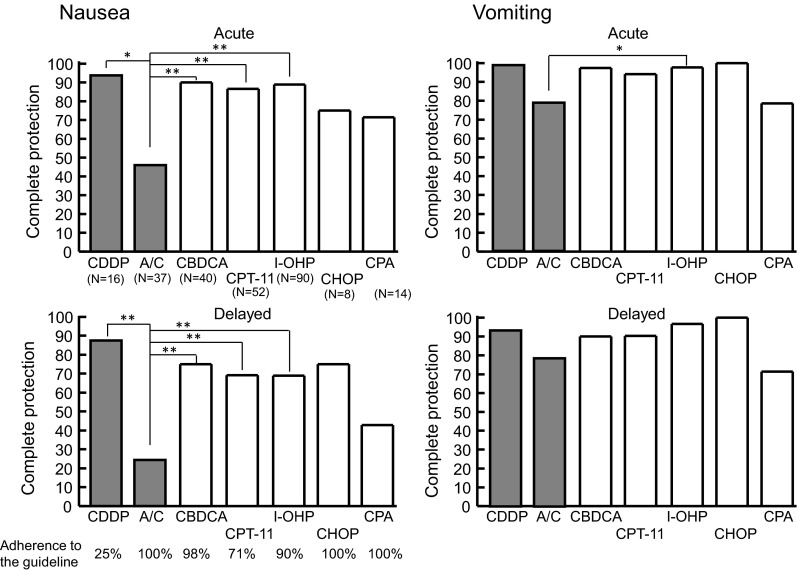
Comparison of the rates of complete protection from nausea and vomiting among various chemotherapy regimens in patients receiving the first cycle of chemotherapy in the outpatient chemotherapy clinic. The number of patients (*n*) is shown in each pair of *parentheses*. Adherence to the Japanese antiemetic guideline during the overall period is represented at *bottom* of figure. *Shaded columns* represent HEC; *open columns* exhibit MEC. *P < 0.05, *P < 0.01 by Kruskal–Wallis test followed by Scheffe’s test

As risk analysis indicated that HEC/MEC and A/C regimen were significant risks for chemotherapy-induced nausea, the rates of complete protection from nausea and vomiting during acute and delayed periods were compared among HEC and MEC regimens in patients who received the first cycle of chemotherapy. As shown in Fig. 2, the rate was significantly lower in A/C regimen (46 % and 24 % for acute and delayed periods, respectively) as compared with other regimens, regardless of 100 % adherence to the guideline-recommended antiemetic medication. In contrast, the control of nausea was favorable for a cisplatin (CDDP)-containing regimen (94 % and 88 % for acute and delayed periods, although the dose of CDDP was low (25 mg/m^2^ in CDDP/gemcitabine for gallbladder cancer or 30 mg/m^2^ in CDDP/irinotecan for gastric cancer).

### Change in the control of nausea and vomiting in A/C regimen after repeated treatment cycles

**Fig. 3:**
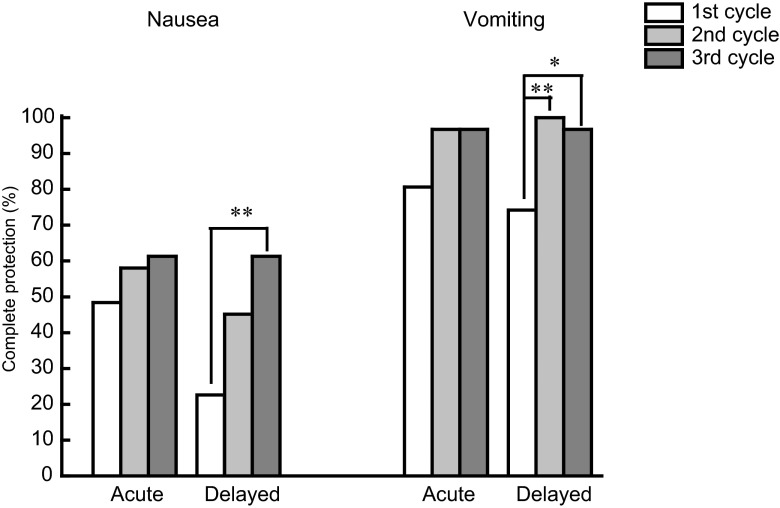
Change in the control of nausea and vomiting in patients receiving three consecutive cycles of anthracycline/cyclophosphamide (A/C) regimen. *P < 0.05, *P < 0.01 by Kruskal–Wallis test followed by Scheffe’s test

Although the complete protection from acute and delayed nausea in the first cycle of A/C chemotherapy was poor (46 % for acute nausea and 24 % for delayed nausea), the rates were improved in the second and third cycles of chemotherapy (Fig. 3). There were significant differences in the rates of delayed nausea and acute as well as delayed vomiting (Kruskal–Wallis test). On the other hand, dose reduction was not carried out in the first cycle in all patients but was performed in one patient in the second cycle (15 % reduction) as well as in the third cycle (15 % reduction).

## Discussion

In the present study, we surveyed the rate of control of CINV in 779 patients who received 5511 cycles of chemotherapy regimens in our outpatient cancer chemotherapy clinic. Among them, patients with gastrointestinal cancer were predominant (51 % of patients and 57 % of chemotherapy cycles).

According to the clinical practice guidelines for antiemesis formulated by ASCO [4], MASCC/ESMO [5], NCCN [6], and JSCO [7], palonosetron, a long-acting second-generation 5-HT_3_ receptor antagonist, is recommended for use in HEC and MEC; however, in the present study, the first generation of 5-HT_3_ receptor antagonists such as granisetron was predominantly prescribed. In the present study, health professionals, including pharmacists and nurses, interviewed all patients and monitored adverse drug reactions. In addition, we checked the prescription for antiemetic medication and aggressively promoted the appropriate use of antiemetic drugs. As a consequence, adherence to the clinical practice guideline for the use of antiemetic drugs was generally high (76 % for HEC, 88 % for MEC, 99 % for low-risk) for patients receiving the first cycle of chemotherapy, except for those undergoing CDDP-containing regimens (25 %). In the CDDP-containing regimens used in the present study, the dose of CDDP was low (25–30 mg/m^2^); thus, aprepitant was excluded from the standard medication for HEC. Even such an antiemetic medication effectively prevented nausea and vomiting, in which the overall control rate was 88 % for nausea and 94 % for vomiting. Very recently, Tamura et al. [19] reported the effectiveness of the antiemetic guideline by a multi-institutional prospective observational study, showing that adherence to the guideline is approximately 74 % for HEC and 95 % for MEC. They also reported that adherence (three antiemetics containing aprepitant, 5-HT_3_ receptor antagonist, and dexamethasone) for HEC decreases the risk for delayed vomiting as compared with two antiemetics (5-HT_3_ receptor antagonist and dexamethasone) without marked influence on the control of nausea. In the present study, the rate of guideline consistency was generally consistent with the data reported by Tamura et al. [19], although the non-adherence did not affect the control of overall nausea or vomiting.

Under the condition of roughly consistent with guideline-recommended antiemetic medication, vomiting was fairly well controlled, but complete protection from nausea was not sufficient for HEC and MEC. The poor control of nausea for HEC in the first cycle occurred primarily in the A/C regimen for breast cancer. Tamura et al. [19] also reported the high incidence of delayed nausea (49.4 % for HEC and 41.7 % for MEC), with a limited incidence of vomiting.

It was notable that the control of nausea and vomiting was generally higher in the overall cycles than in the first cycle. Particularly, marked improvement of the control rate of delayed nausea was observed at the second and third cycles of A/C chemotherapy. It is unlikely that improvement of the control of nausea results from decrease in the dose of anticancer drugs because, in the present study, the dose reduction was not observed in the first cycle but was carried out in one patient in the second cycle (15 % reduction) as well as in the third cycle (15 % reduction). Patients who showed failure in the control of CINV in the previous cycle were administered prochlorperazine or olanzapine in addition to an antianxiety drug such as lorazepam in the following cycles, which may be the predominant reason for the improvement of CINV control in the subsequent cycles. Moreover, in 608 patients who received the first cycle of HEC, MEC, low, or minimal risk of chemotherapy, the dose intensity was quite similar between patients with and without complete protection from nausea, in which the values were 97.0 ± 8.1 % (mean ± SD) in 450 patients who showed complete protection from nausea and 97.0 ± 7.5 % in 158 patients who did not. In addition, the complete protection from nausea was 71.4 % (60 of 84 patients) in patients with dose reduction and 74.4 % (390 of 524 patients) in those without dose reduction (*P* = 0.654 by *χ*^2^ test). Therefore, it is unlikely that the dose of anticancer drugs affected the control of chemotherapy-induced nausea in the present study.

Several investigators have shown the risk factors for CINV. Female gender, age, no history of drinking, and history of hyperemesis gravidarum are common as risks that lead to a loss of control of CINV [19–26]. Sekine et al. [21] showed in patients receiving HEC or MEC that female gender has high risk (OR 2.49) for failure in complete response (no vomiting and no rescue). It has also been reported that female patients are more likely to experience nausea and vomiting than male patients receiving HEC or MEC [22, 23]. On the other hand, Hesketh et al. [24] reported in patients receiving CDDP (≥70 mg/m^2^) that females are at high risk for the inability to complete response (OR 1.303) only when aprepitant is not included in the antiemetic medication. Younger age is also a risk for the loss of emetic control, but the cutoff age differs among studies, ranging from 40 to 65 years old [20–23]. Tamura et al. [19] also reported that older age is a decreased risk for CINV. However, the cutoff value of age that influences the control of CINV is still unclear. In the present study, the cutoff value of age was estimated from the ROC curve method, in which the AUC was 0.658 for nausea (low accuracy prediction) and 0.721 for vomiting (moderate accuracy prediction). The cutoff age was 58.5 years old as determined by the Youden index or 61.5 years old by the distance method and was set at 60 years old. Interestingly, the cutoff age for vomiting predicted by Youden index and distance method (49.5 years old) was younger than that for nausea. As a consequence, age under 60 years old was a significant risk for nausea (OR 2.303; 95 % CI, 1.525–3.477, *P* < 0.001), whereas age under 50 years old was a significant risk for vomiting (OR 5.803; 95 % CI, 2.667–12.63, *P* < 0.001). In addition, female gender (OR 1.615; 1.022–2.552, *P* = 0.04 for nausea; OR 3.151; 1.213–8.183, *P* = 0.018 for vomiting) and HEC/MEC (OR 2.321; 1.489–3.617, *P* < 0.001 for nausea; OR 2.993; 1.245–7.195, *P* = 0.014 for vomiting) were also significant risks for nausea or vomiting, although A/C chemotherapy was a significant risk for nausea but not for vomiting (OR 4.955; 1.863–13.18, *P* = 0.001). Our present findings indicating the difference in the cutoff age between nausea and vomiting suggest that the differences in the cutoff age among studies may result from different indices of the control of CINV, including complete response, complete control, and complete protection from nausea or vomiting.

These findings, taken together, suggested that extensive antiemetic medication using other types of antiemetic drugs such as olanzapine in addition to the standard medication is required for prevention of chemotherapy-induced nausea in patients who possess risks for poor control of CINV, including being female, younger age, and A/C chemotherapy.

In the present study, no marked difference in the control of CINV among MEC except for cyclophosphamide-base regimens other than the A/C regimen, although there was a marked difference in the control of CINV among HEC, as mentioned earlier. The low rate of the control of CINV for cyclophosphamide-base regimens may be caused by the patient risks (female and young age) rather than the chemotherapy, because the cyclophosphamide-base regimens were used for the most part in breast cancer patients, whose average age was 48 years.

In conclusion, the control of CINV was investigated in 779 patients receiving 5511 cycles of chemotherapy regimens in our outpatient cancer chemotherapy clinic. In spite of the high rate of adherence to the antiemetic guideline, the control of nausea, but not vomiting, was poor in patients receiving HEC and MEC. A multivariate logistic regression analysis indicated that female gender, age under 60 years, HEC/MEC, and A/C chemotherapy were significant risks for overall nausea. Care should be taken to prevent chemotherapy-induced nausea in high-risk patients.
